# Perfusable micro-vascularized 3D tissue array for high-throughput vascular phenotypic screening

**DOI:** 10.1186/s40580-022-00306-w

**Published:** 2022-04-08

**Authors:** James Yu, Somin Lee, Jiyoung Song, Seung-Ryeol Lee, Suryong Kim, Hyeri Choi, Habin Kang, Yunchan Hwang, Young-Kwon Hong, Noo Li Jeon

**Affiliations:** 1 Interdisciplinary Program in Bioengineering, Seoul National University, 1 Gwanak-ro, Gwanak-gu, Seoul, 08826 Republic of Korea; 2 Department of Mechanical Engineering, Seoul National University, 1 Gwanak-ro, Gwanak-gu, Seoul, 08826 Republic of Korea; 3 Department of Electrical Engineering and Computer Science, Seoul National University, 1 Gwanak-ro, Gwanak-gu, Seoul, 08826 Republic of Korea; 4 Department of Surgery, Norris Comprehensive Cancer Center, Keck School of Medicine, University of Southern California, Los Angeles, CA 90033 USA; 5 Institute of Advanced Machines and Design, Seoul National University, 1 Gwanak-ro, Gwanak-gu, Seoul, 08826 Republic of Korea

**Keywords:** Vascularized micro tissue, Organ-on-a-chip, Angiogenesis, Sequential edge guided patterning, Microfluidics

## Abstract

Microfluidic organ-on-a-chip technologies have enabled construction of biomimetic physiologically and pathologically relevant models. This paper describes an injection molded microfluidic platform that utilizes a novel sequential edge-guided patterning method based on spontaneous capillary flow to realize three-dimensional co-culture models and form an array of micro-vascularized tissues (28 per 1 × 2-inch slide format). The MicroVascular Injection-Molded Plastic Array 3D Culture (MV-IMPACT) platform is fabricated by injection molding, resulting in devices that are reliable and easy to use. By patterning hydrogels containing human umbilical endothelial cells and fibroblasts in close proximity and allowing them to form vasculogenic networks, an array of perfusable vascularized micro-tissues can be formed in a highly efficient manner. The high-throughput generation of angiogenic sprouts was quantified and their uniformity was characterized. Due to its compact design (half the size of a 96-well microtiter plate), it requires small amount of reagents and cells per device. In addition, the device design is compatible with a high content imaging machine such as Yokogawa CQ-1. Furthermore, we demonstrated the potential of our platform for high-throughput phenotypic screening by testing the effect of DAPT, a chemical known to affect angiogenesis. The MV-IMPACT represent a significant improvement over our previous PDMS-based devices in terms of molding 3D co-culture conditions at much higher throughput with added reliability and robustness in obtaining vascular micro-tissues and will provide a platform for developing applications in drug screening and development.

**Supplementary Information:**

The online version contains supplementary material available at 10.1186/s40580-022-00306-w.

## Introduction

Vasculature exists as an integral feature of all organs within the human body. As the primary component of systemic circulation, vasculature facilitates far-reaching functions ranging from oxygen and nutrient delivery, waste removal, and immune cell migration and cancer metastasis. Investigations on vascular biology can provide physiological and pathological conditions as well as drug safety/efficacy testing and development of therapeutics [1–9]. Conventional methods for pre-clinical modelling of human vasculature, and screening for therapeutics efficacy use animal models and 2D in vitro cell culture systems [4, 10, 11]. Advances in microfabrication technologies, as well as ever-present concerns regarding model organism to human translatability, and ethical considerations for in vivo animal experimentation has generated demand for sophisticated and higher throughput in vitro biomimetic test platforms [6, 10, 12–22].

Micro-Physiological Systems (MPS), often associated with microfluidic tissue-on-a-chip and organ-on-a-chip systems, are in vitro platforms for engineering in vivo like micro-environmental conditions as a means to culture physiologically relevant tissue-like morphologies from in vitro cell cultures beyond those generated from conventional 2D adherent cell cultures [10, 23]. MPS models for angiogenesis, the sprouting of new blood vessels from pre-existing large vessels, and vasculogenesis, the development of nascent blood vessels from disorganized endothelial cells, are sought-after tools in vascular biological research [5, 10, 11, 16, 24]. Conventional in vivo and ex vivo assays like the retinal whole mount, and zebrafish models have served in experimental niches similar to that of in vitro MPS based vessel formation assays, as a means of functionally assaying vessel morphologies at a tissue level, but with the same limitations of in vivo experimentation [4, 12–14, 25]. Given the foundational role of vasculature in the human body, models can range broadly from simple endothelial cell network formation assays to sophisticated models of cancer metastasis in perfusable vascularized tissues [26–30]. Vascular MPS models see potential applications incorporating high throughput in “pre-preclinical” drug testing, pathology modelling, vascular bio mechanistic studies, and more [10, 31, 32].

Microfluidic tissue culture device schemes vary widely, ranging from substrates such as polydimethylsiloxane (PDMS) to polystyrene (PS), media exchange methods from passive levelling flow to active pump systems, and fluid patterning methods such as hydrophobic capillary burst patterning to hydrophilic spontaneous capillary flow (SCF) [10, 17, 33]. Despite the wide variety of highly distinct designs for angiogenesis/vasculogenesis MPS platforms, most operate under a common set of engineering constraints and features inherent to the biology behind micro physiological conditions reconstituted—for instance, robust mechanisms for patterning controllable dimensions of cell-laden and acellular hydrogels to reconstitute bulk tissues, cell monolayers as endothelial vessel wall analogs, and the induction of trans-endothelial and shear forces to emulate fluid pressure and flow conditions physiologically relevant to vascular tissues [10, 34, 35]. While the fundamental operating principles behind vascular MPS platforms may be conserved, there is a growing significant divide between conventional and next generation systems lies in fabrication methods, designs, and substrates of the platforms themselves.

Recent efforts to address the scalability and accessibility of microfluidic MPS platforms have seen the development of standardized polystyrene (PS) based injection moldable microfluidic devices for mass production [20, 33, 36]. In contrast to conventional lithography-based fabrication methods, injection molding affords several key fabrication design freedoms which allow for the centralized mass production of devices and the comparatively trivial incorporation of vertically integrated designs for more space efficient multilevel form factors [33, 37]. Injection molded MPS platforms, as a necessary step towards commercially accessible systems, also seek to reduce complexity in order to maximize production efficiency, ease of use, and decrease device-to-device variability while retaining the capability to conduct both straightforward mass quantitative assays as well as more sophisticated functional assays [37]. Such improvements are necessary to open accessibility of microfluidic tissue culture as a tool for biologists and industry at large.

The incorporation of open microfluidics and hydrophilic surface induced spontaneous capillary patterning (SCP) of droplets have also emerged as a means to increase the accessibility of microfluidic devices [33, 34]. Droplet patterning technologies serve to simplify the macro/micro interface between user and channel by removing the need for narrow injection ports and positive pressure loading, and allowing for the use of automated and electronic pipettes for faster and more uniform patterning [33, 36].

In this work, we present an injection molded microtiter plate format platform for 3D Angiogenic Sprouting and Perfusable Vascular Network assays (MV-IMPACT). We demonstrated the versatility of the platform to conduct simple quantitative angiogenesis assays as well as complex qualitative assays to model cancer-vascular interactions with regards to tumor intra and extravasation.

## Experimental section/methods

### Fabrication

Polystyrene (PS) injection molding was performed at R&D Factory (Korea). The aluminum alloy mold core was processed by machining and polishing. The clamping force at the time of injection was set to 130 ton with a maximum injection pressure of 55 bar, 15 s of cycle time, and a 220 °C nozzle temperature. A PS substrate plate was then subsequently bonded to the upper bulk chip via ultrasonic welding (Branson, USA).

### Cell preparation

Human umbilical endothelial cells (HUVECs; Lonza, Swiss) were cultured in endothelial growth medium 2 (EGM-2; Lonza). Normal Lung fibroblasts (nLFs; Lonza) were cultured in fibroblast growth medium 2 (FGM-2; Lonza). Red fluorescent protein (RFP) and green fluorescent protein (GFP)-labelled HUVECs were procured at P3 and subcultured to produce P6 frozen stock. All utilized tumor cell lines (KCLB, Korea) were cultured in RPMI-1640 (Gibco, USA). The cells were incubated at 37 °C in 5% CO_2_ for 3 days prior to loading. Cultured LFs, HUVECs, and tumor cell lines were detached from the culture dish using 0.25% Trypsin–EDTA (HyClone, USA). The various cells were then re-suspended in bovine fibrinogen solutions at the concentrations required for each experimental condition.

### Hydrogel and cell patterning

Prior to device seeding, all chips were plasma surface treated at 70 W for 3 min to induce surface hydrophilicity (Femto Science, Korea). Fluid patterning within the device was done via patterning the center channel, then the side channels as needed. Center channel patterning was done by priming 25 µL of a cellular or acellular bovine fibrinogen solution (final concentration 2.5 mg mL^−1^; Sigma, USA) with 1 µL of bovine thrombin (0.25 U mL^−1^; Sigma). Next, 1 µL of the cellular or acellular fibrinogen and thrombin mixture was injected along the inner edge of the well plate, where the capillary forces spontaneously selectively patterned the gel into the central channel. The primed hydrogel mixture was then allowed to crosslink for 5 min before subsequent channels were patterned. Up to 14 unit device center channels were patterned at a time utilizing an electronic pipette (AND, Japan). Side channels were patterned by mixing 75 µL of a cellular or acellular bovine fibrinogen solution (final concentration 2.5 mg mL^−1^) with 2 µL of bovine thrombin (0.5 U mL^−1^). The cellular or acellular fibrinogen and thrombin mixture was then dispensed 3 µL at a time along the inner edge of the well plate, where capillary forces spontaneously and selectively pattern the side channels adjacent to the already patterned central gel. The primed hydrogel mixture was then allowed to crosslink for 3 min before the media reservoirs were filled with medium, or for the patterning of the other side channel with a total of three hydrogel patterns. With the use of electronic pipettes, up to 14 side channels were patterned at a time per given primed gel mixing. When patterning cell suspensions for monolayer seeding, suspension of cells in either PBS or EGM-2 media were patterned 3 µL at a time onto the side channels, and the chips were rotated such that the cell suspension laden side channels were directly above the gel patterned central channel to facilitate cell settling by gravity. Monolayer seeding chips were incubated between 5 and 30 min rotated on to the side at 37 °C to produce confluent monolayers. When adding media, up to 100 µL of EGM-2 (or relevant media) was dispensed via multi-channel or electronic pipette per reservoir. Flow conditions could be induced by adding more media to one of the two reservoirs, and/or tilting the chip to the side to raise the fluid level of one reservoir over the other.

### High throughput fluid handling

All of the hydrogel patterning carried out during the course of this study was done via electronic pipette, which allowed for the simultaneous patterning of up to 14 replicate wells patterned by a single person per minute long patterning session, with minimal user-to-user variability due to automatic droplet patterning and a high tolerance open-channel patterning mechanism. All aspiration and dispensing associated with media change, fixation, immunostaining were done via multichannel aspirators and pipettes which allowed a single person to completely aspirate and dispense media for an entire 28 replicate well chip within minutes.

### Drug treatment

DAPT (SIGMA, USA) was prepared as a 10 mM stock solution in DMSO, then brought to a final concentration of 25 µM DAPT and 0.25% DMSO by volume in EGM-2 (Lonza). Vehicular control media constituted EGM-2 with 0.25% DMSO by volume, while untreated control media utilized pure EGM-2.

### Immunocytochemistry

Co-cultured tissues in the device were fixed by aspiration of both reservoirs followed by 20 µL of 4% (w/v) paraformaldehyde (Biosesang, Korea) in PBS (Gibco, USA) in one reservoir per unit well with a 15 min incubation time bench top. Post fixation, both reservoirs were aspirated and optionally stored in 50 µL of dPBS per reservoir until ready for staining, or stained directly. Endothelial cell (EC) specific vessel staining was done with 488 fluorescein-labelled Ulex Europaeus Agglutinin I (Vector, UK), which was prepared at a 1:1000 ratio of dye in dPBS. Per unit well, 30 µL of dye solution was added to one reservoir and 10 µL to the other in order to facilitate the flow of dye from one reservoir, through the tissues, to the other. Samples were incubated at 4 °C overnight, then stored in 100 µL of dPBS per reservoir per unit well for imaging. Imaging was performed utilizing spinning disk confocal microscopy (Yokogawa CQ-1, Japan), and epifluorescence microscopy (Nikon TI-2, Japan) to produce to produce 3D and z-stackable images of the angiogenic and vasculogenic networks for figure generation and quantitative analysis of vasculature.

Given the standardized 384 well microtiter plate form factor of the platform, we utilized automated acquisition scripts to image entire 28 device chips utilizing imaging macros within both the Nikon and Yokogawa system.

### Statistical analysis

Fiji (http://fiji.sc.), open access software, was used to analyze confocal images of vasculature or tumor. All statistical analyses performed unpaired two-tailed Student’s t-test to obtain statistical comparisons of analyzed values. The p value thresholds for statistical significance were set and represented in the graph as *p < 0.05; **p < 0.005; ***p < 0.0005; ****p < 0.00005.

Angiogenesis Analyzer was used to process DAPT treated angiogenesis screening images as a means to automatically acquire skeletonize sprouts and count branching to derive mean vessel width, branch numbers, and mean vessel length values [38].

## Results and discussion

### Design and function of the MV-IMPACT platform

**Fig. 1:**
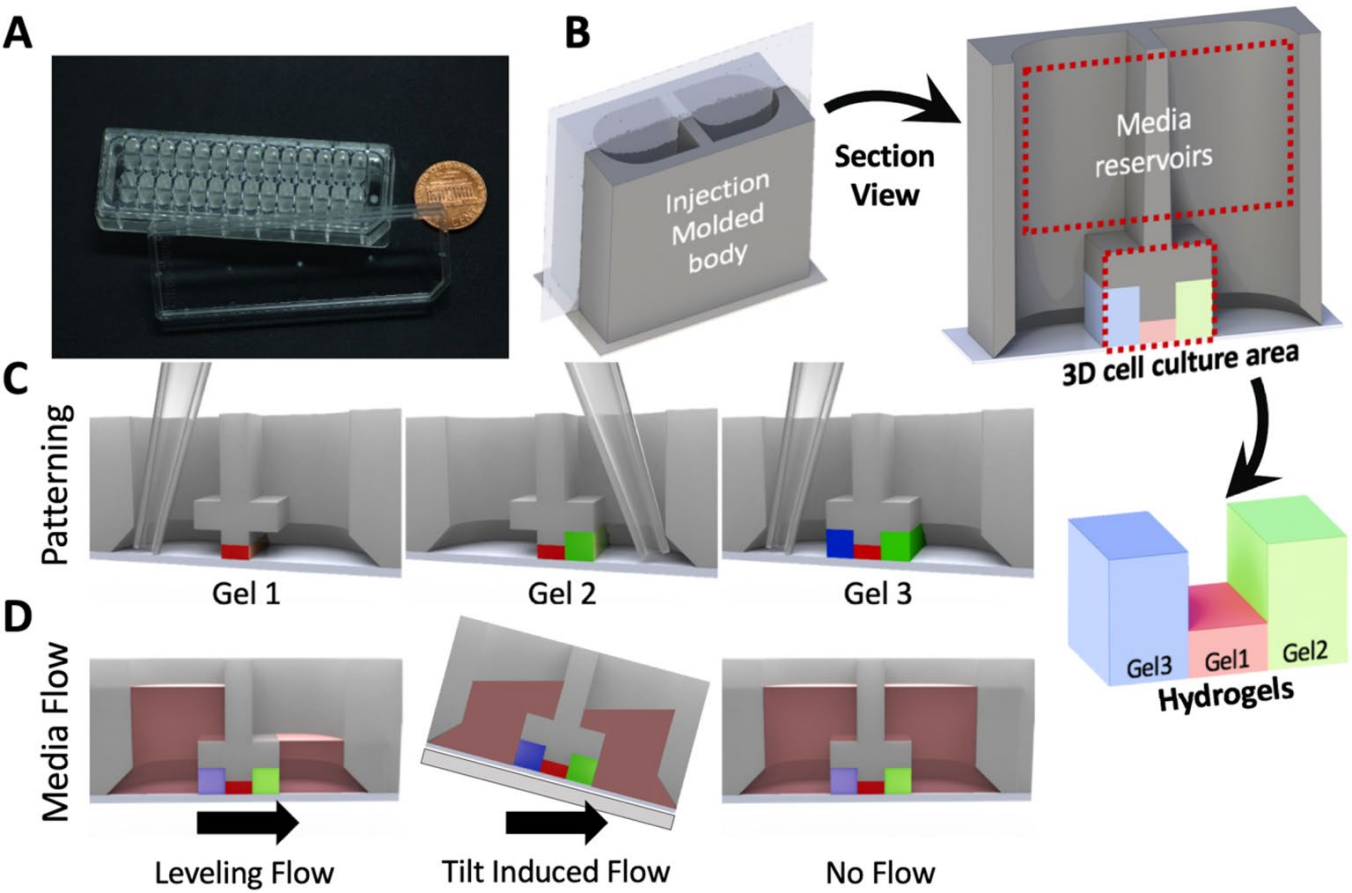
Overview of chip design and hydrogel/media patterning. **A** A photograph of the MV-IMPACT chip body with top lid. **B** Rendering of a single unit well with cross section view of the entire well and patterned gel. **C** Illustration of edge guided sequential patterning within a cross section view of a unit well. **D** Different methods of inducing flow conditions. From left to right, levelling flow utilizes differences in media volumes within the two reservoirs to induce flow through the patterned gels via equilibration. Tilt induced flow elevates one reservoir over another to facilitate flow. Static flow conditions can be induced by adding the same volume of media in both reservoirs

MV-IMPACT consists of single injection molded polystyrene (PS) body housing the microfluidic patterning geometries and the media reservoir, adhesive bonded to a polycarbonate (PC) film substrate, with an optional injection molded PS cap (Fig. 1A). Each unit well consists of two 384 well plate format wells which serve as two separate media reservoir compartments which connect at the hydrogel patterning regions at the interface between the film substrate and the bottom of the main body (Fig. 1B). To facilitate standardized form factor compatibility, the device complies with the 384 well microtiter plate form factor for direct 4.5 mm center-to-center handling, and is also compatible with staggered fluid handling with 9 mm center-to-center 96 well plate handling infrastructure.

Compared to equivalent conventional soft lithographic PDMS based platforms, the mass produced injection molded MV-IMPACT is capable of much higher throughput in terms of size and operation, and is the most compact multi-channel platform for 3D angiogenesis to date.

**Fig. 2:**
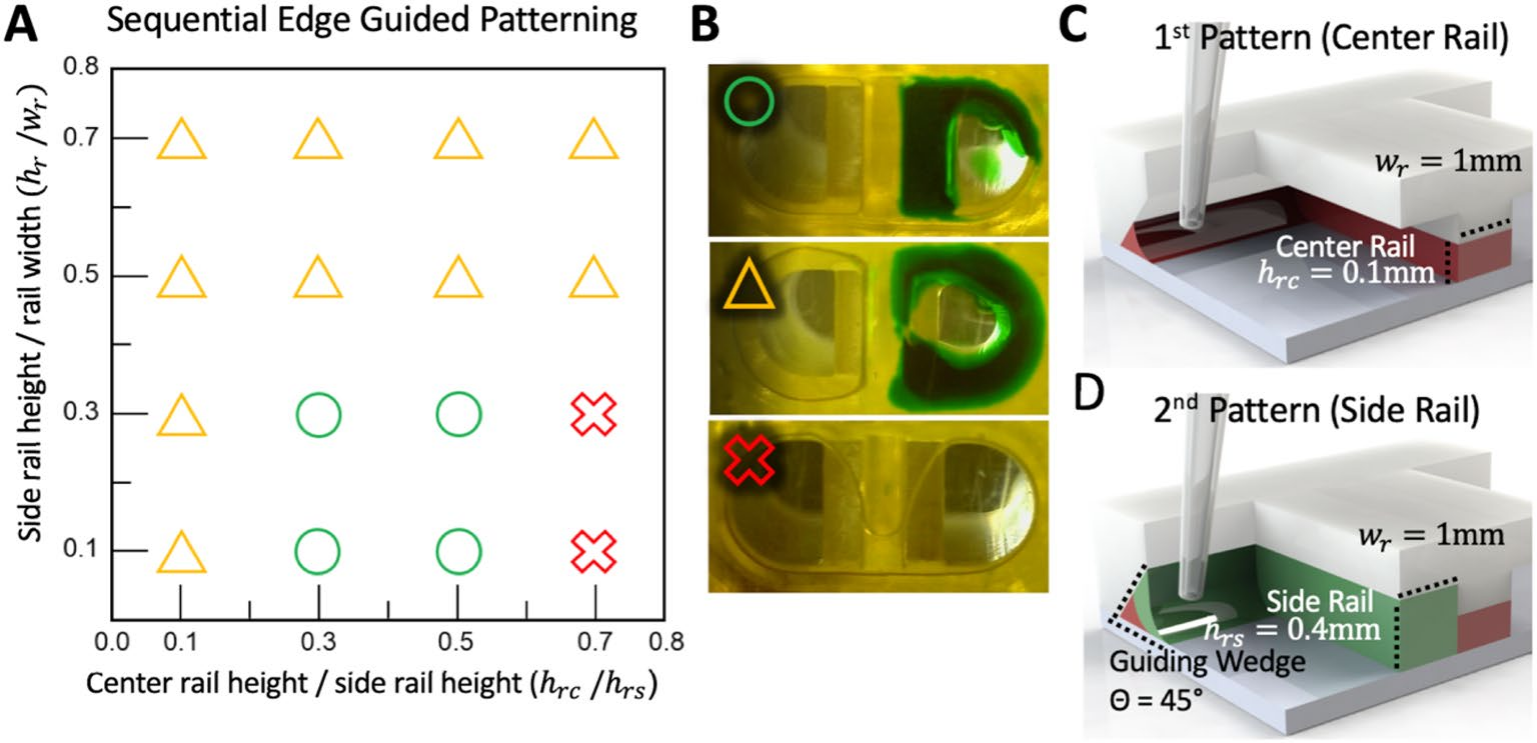
Detailing the specifics of the novel sequential edge guided patterning mechanism. **A** A graph of the solution space of dimensions required for utilizing the wedge to rail capillary flow mechanism to sequentially pattern a primary central and secondary side rail utilizing the same wedge, for up to three (one central and two side) patterns within a unit chip. **B** Illustrates the success and failure conditions for patterning. The green circle denotes a successful 1st center rail and 2nd side rail patterning. The yellow triangle shows a successful 1st center rail patterning, but a failed 2nd side rail pattern due the inability for the 2nd pattern to fill the side rail. The red x denotes a 1st center rail pattern failure. **C** Shows a rendering of a successful 1st center rail patterning utilizing the optimized dimensions of the platform. **D** Shows a rendering of a successful 2nd side rail patterning utilizing the optimized dimensions of the platform

The MV-IMPACT utilizes air plasma induced hydrophilic surface modification to facilitate spontaneous capillary flow patterning (SCP) of droplets (Fig. 2C). Differences in patterning rail heights allow for the selective and sequential patterning of hydrogels and other fluids, reusing liquid wedges to simplify loading and remove the need for cumbersome, low tolerance injection ports for loading (Fig. 2D).

**Fig. 3:**
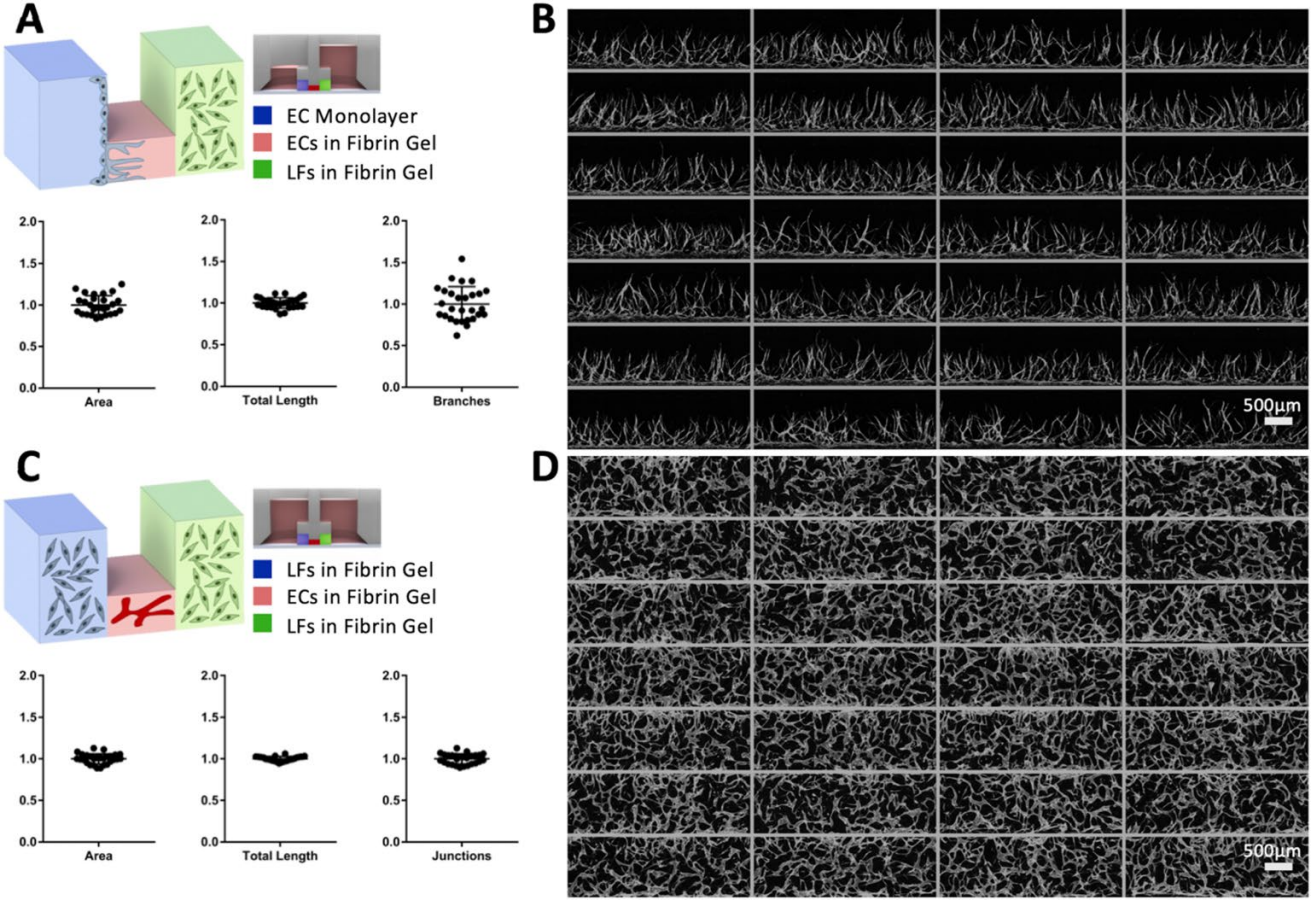
Overview angiogenesis and vasculogenesis patterning configurations and sample output. **A** Schematic diagram of an angiogenesis assay. 1 µL of acellular fibrin gel is patterned in the central lane (red), followed by 2.5 µL of lung fibroblasts in fibrin gel (green) in the right side lane, and 2.5 µL of HUVEC suspension deposited as a confluent monolayer is seeded in the left (blue) side lane. Flow from the LF to EC compartments is induced by adding media to the LF side reservoir. Adjusted standard deviations for Area, Total Length, and Branches are: 0.1118, 0.0626, and 0.2047, respectively (n = 28). **B** Sample 28 well imaging output of angiogenesis within a single injection molded chip, center lane (red) was selected as ROI. **C** Schematic diagram of a vasculogenesis assay. 1 µL of HUVECs suspended in fibrin gel is patterned in the central lane (red), followed by 2.5 µL of LFs in fibrin gel in both the right (green) and left (blue) side lanes. Adjusted standard deviations for Area, Total Length, and Junctions are: 0.0596, 0.0276, and 0.0594, respectively (n = 28). **D** Sample 28 well imaging output of vasculogenesis within a single injection molded chip, center lane (red) was selected as ROI. The measurement was performed after 7 days of cultivation

The flexible nature of hydrogel and cell suspension seeding (Fig. 1B) and media flow conditions (Fig. 1C) allows for a highly modular cell seeding capability with dynamic and static media conditions with a high degree of patterning uniformity in both angiogenesis and vasculogenesis assays (Fig. 3A, B).

### Sequential edge guided patterning

The concept of patterning liquid from wedge to rail has been introduced in a previous study. [33] Lee et al. established a design rule for single-use pattern guidance along a surface perpendicular to the substrate (90°) to a patterning rail. The MV-IMPACT platform utilizes an acute angled surface to sequentially pattern fluids along the same surface to several rails, starting with rails with smaller height (Fig. 2C, D). Under hydrophilic surface conditions, fluid tends to flow along a pressure gradient set from wider to narrower capillaries [39, 40]. The Concus-Finn equation also stipulates that the smaller the wedge angle in the hydrophilic state, the larger the range of contact angles and the better the liquid flows along the wedge. Based on this phenomena, the platform was designed to have the sharp wedge and different height of the rails. Specifically, the wedge angle is 45° and the height of the side rails were set higher than that of the center rail. The height of the wedge is designed to be equal to the height of the side rail. As a result, the first patterning proceeds from the wedge to the center rail and the second patterning proceeds from the wedge to the side rail.

A parametric study was conducted to determine the design rule when the wedge angle was 45° (Fig. 2). The experiment was performed with varying the height of center and side rail under the same width of 1 mm for both rails: height of side rail (hrs) and wedge rail (hw) and height of center rail (hrc), where hrs is equal to hw. Each design was fabricated by 3D printer and liquid patterning was performed. The fibrin gel was used for the first pattering, while the green dye was used for second patterning. When the height difference between rails is small, the first patterning fails without filling center and side rails separately. On the contrary, when the height difference is large, the first patterning succeeds but the second patterning fails. In particular, when hw exceeds 1 mm, the secondary patterning fails. From this experiment, the design rule was roughly established. We adopted 0.1 mm height for the center rail, and 0.4 mm height for the side rail with 1 mm width.

### Vasculogenic and angiogenic tissue culture

The MV-IMPACT platform is capable of a variety of different cell seeding configurations to suit specific assay types. Angiogenesis, the sprouting of new vessels from a pre-established larger vessel, can be accomplished by patterning 1 µL of acellular fibrin gel in the central lane, followed by 2.5 µL of lung fibroblasts suspended in fibrin gel in the right side lane, and 2.5 µL of HUVEC suspension deposited as a confluent monolayer is seeded in the left side lane. Flow from the LF to EC compartments is induced by adding media to the LF side reservoir. To assess the variability in the outputs of top-down cross sectional area, total vessel length, and branches all values for each metric were normalized around the mean. Adjusted standard deviations for Area, Total Length, and Branches are: 0.1118, 0.0626, and 0.1634, respectively (Fig. 3A, B).

Vasculogenesis, the formation of nascent blood vessels, can be assayed by patterning 1 µL of HUVECs suspended in fibrin gel in the central lane, followed by 2.5 µL of LFs in fibrin gel in both the right and left side lanes. To assess the variability in the outputs of top-down cross sectional area, total vessel length, and total number of junctions, all values for each metric were normalized around the mean. Adjusted standard deviations for Area, Total Length, and Junctions are: 0.0596, 0.0276, and 0.0594, respectively.

### Cancer angiogenesis assay

The mechanisms by which cancers establish vascular networks to supply bulk tumor tissues are poorly understood, and high throughput platforms to quantify angiogenic performance of cancer cell types within controllable test groups are a necessary step towards in vitro studies into cancer angiogenesis.

**Fig. 4:**
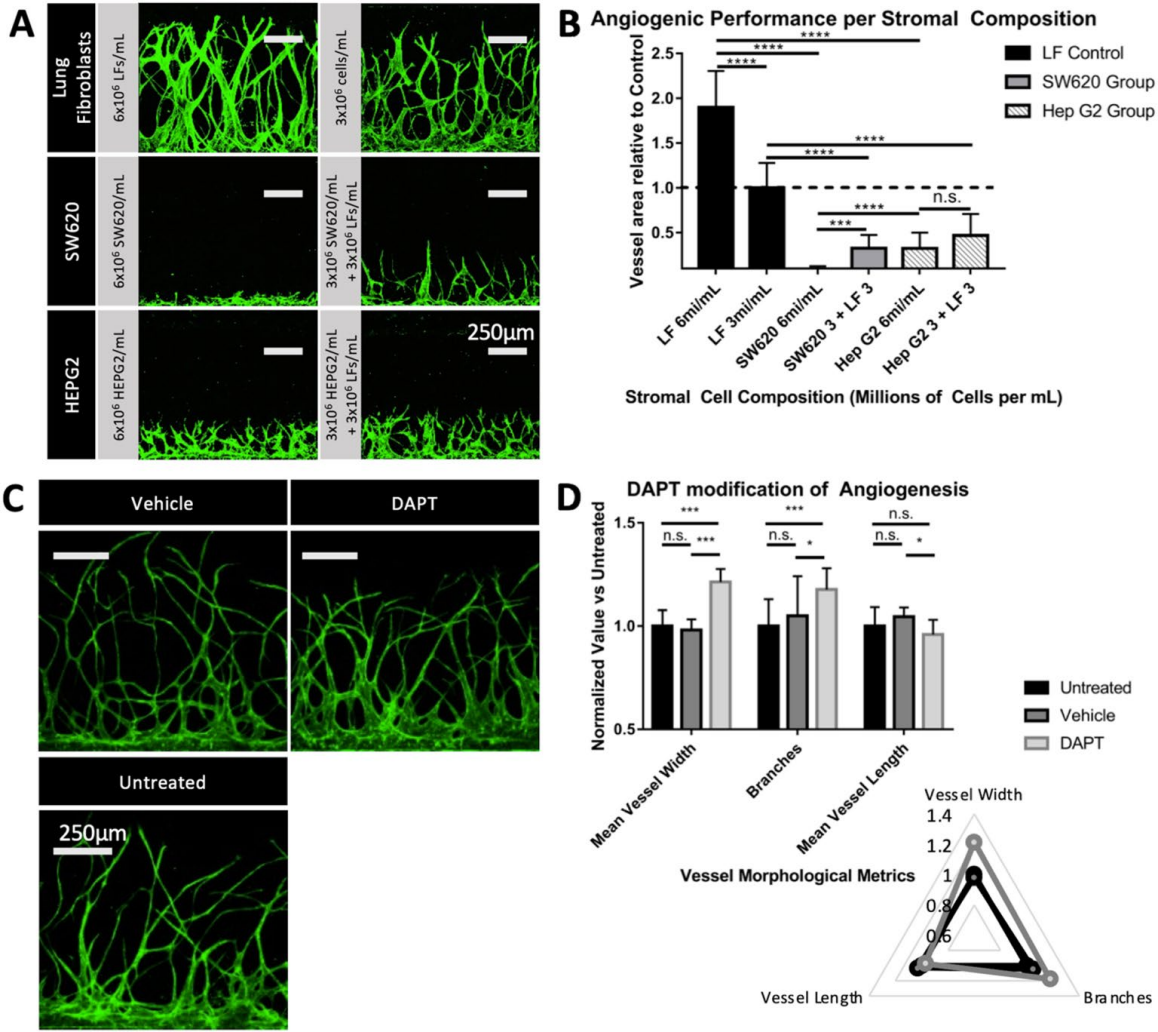
Angiogenic assays conducted testing for stromal component mediated and DAPT induced notch inhibition mediated morphological differences. **A** Confocal images of angiogenic sprouting induced by stromal cell component groups comprising of normal human lung fibroblasts at 6 × 10^6^ cells mL^−1^ and 3 × 10^6^ cells mL^−1^ seeding concentrations, 6 × 10^6^ cells mL^−1^ SW620, 3 × 10^6^ cells mL^−1^ SW620 + 3 × 10^6^ cells mL^−1^ LF, 6 × 10^6^ cells mL^−1^ HEPG2, and 3 × 10^6^ cells mL^−1^ HEPG2 + 3 × 10^6^ cells mL^−1^ LF. **B** Charts the top down vessel areas relative to the 3 × 10^6^ cells mL^−1^ LF control. **C** Confocal images of angiogenic sprouting under untreated control (EGM-2 media only), DMSO control (EGM-2 media with 0.25% DMSO by volume), and DAPT treatment (EGM-2 media with 25 µM DAPT and 0.25% DMSO by volume). **D**, **E** Chart visualizing mean vessel width, branching, and mean vessel length between treatment groups. The quantification of angiogenic sprouting was performed after 7 days of cultivation within the chip (*p < 0.05; **p < 0.005; ***p < 0.0005; ****p < 0.00005 and n.s. not significant)

To benchmark the concentration and composition dependent angiogenic performance of cancer and normal stromal cells, compositions of normal human Lung Fibroblasts (LF) at 6 × 10^6^ cells mL^−1^ and 3 × 10^6^ cells mL^−1^, human colorectal adenocarcinoma SW620 and human hepatocellular carcinoma (HepG2) cancer cell lines at 6 × 10^6^ cells mL^−1^, and mixtures of each respective cancer cell line with equal final concentrations of LFs to consist of 3 × 10^6^ cells mL^−1^ HepG2 + 3 × 10^6^ cells mL^−1^ LF, and 3 × 10^6^ cells mL^−1^ SW620 + 3 × 10^6^ cells mL^−1^ – for a 6 × 10^6^ cells mL^−1^ total end concentration of stromal cells for all but the 3 × 10^6^ cells mL^−1^ LF group. The experiment was arranged in such a way that directly compares stromal cell types at 6 × 10^6^ cells mL^−1^ (6 × 10^6^ cells mL^−1^ LF only, 6 × 10^6^ cells mL^−1^ HepG2 only, and 6 × 10^6^ cells mL^−1^ SW620 only groups), and potential interactive effects of heterogeneous stromal components between the cancer and LF intermix groups compared with controls with same final stromal cell concentration (6 × 10^6^ cells mL^−1^ LF, 3 × 10^6^ cells mL^−1^ HEPG2 + 3 × 10^6^ cells mL^−1^ LF, and 3 × 10^6^ cells mL^−1^ SW620 + 3 × 10^6^ cells mL^−1^ LF), and a comparison with a group consisting of the same amount of LFs (3 × 10^6^ cells mL^−1^ LF only). In all groups, chips were loaded in the angiogenesis assay configuration as shown in Fig. 3A, consisting of an acellular 2.5 mg mL^−1^ fibrin hydrogel seeded with 6 × 10^6^ cells mL^−1^ HUVEC monolayer on one side, and 2.5 mg mL^−1^ fibrin hydrogel laden with stromal cells on the other. The HUVEC angiogenic sprouts into the hydrogel from the HUVEC monolayer towards the stromal channel were imaged, flattened, and quantified for area as shown in Fig. 4A and B. The results were as follows: LF 3 × 10^6^ cells mL^−1^ was defined as an area reference control with a mean area of 1 and an SD of 0.212; LF 6 × 10^6^ cells mL^−1^ mean area 1.684 (as a proportion of the LF3 × 10^6^ cells mL^−1^ control), SD 0.308; SW620 6 × 10^6^ cells mL^−1^ area 0.239, SD 0.096; SW620 3 × 10^6^ cells mL^−1^ + LF 3 × 10^6^ cells mL^−1^ area 0.488, SD 0.113; HEPG2 6 × 10^6^ cells mL^−1^ 0.486, SD 0.134; HEPG2 3 × 10^6^ cells mL^−1^ + LF 3 × 10^6^ cells mL^−1^ area 0.596, SD 0.123.

The angiogenic potential of each stromal composition varied significantly in all but one test groups.

In the assay between Lung Fibroblasts at 6 × 10^6^ cells mL^−1^ and LFs at the reference concentration of 3 × 10^6^ cells mL^−1^, LF 6 × 10^6^ cells mL^−1^ exhibited 1.684 times the angiogenic sprouting performance of the control (p = 9.404 × 10–6), establishing a significant relationship between higher concentrations of a given pro-angiogenic stromal composition and angiogenic performance.

Comparing the angiogenic potential of cancer cell lines at 6 × 10^6^ cells mL^−1^ concentrations vs LF 6 × 10^6^ cells mL^−1^ control, SW620 and HEPG2 performed at 0.142 (p = 4.126 × 10–9) and 0.289 (p = 1.876 × 10–8) times the area of 6 × 10^6^ cells mL^−1^ LF control, respectively. This indicates that the SW620 and HEPG2 cell line stocks used exhibited lower pro-angiogenic potential than that of the Lung Fibroblast control at the same seeding concentration.

Between heterogeneous stromal components consisting of 3 × 10^6^ cells mL^−1^ cancer mixed with 3 × 10^6^ cells mL^−1^ LFs, the total cell concentration control of 6 × 10^6^ cells mL^−1^ LF, and the 3 × 10^6^ cells mL^−1^ LF only control, SW620 + LF exhibited lower performance against both (0.488 vs 3 × 10^6^ cells mL^−1^ LF, p = 1.466 × 10–6) (0.29 vs 6 × 10^6^ cells mL^−1^ LF, p = 4.126 × 10–9), and HEPG2 + LF did the same (0.596 vs 3 × 10^6^ cells mL^−1^ LF, p = 2.18 × 10–8) (0.354 vs 6 × 10^6^ cells mL^−1^ LF, p = 2.18 × 10–8). The lower angiogenic performance of the cancer and LF mixed stromal components vs that of the 3 × 10^6^ cells mL^−1^ LF control indicates a potential anti-angiogenic interaction between the tested cell lines and normal fibroblasts, and bears more in-depth biomechanical investigation. Further work investigating the angiogenic potential of normal and cancer associated fibroblasts in co-culture with tumor cells in this platform is currently underway at this time.

### DAPT induced vascular morphology characterization

The applicability of the MV-IMPACT platform to high throughput and high content quantification of vessel morphology in response to drug treatment was assessed through the use of angiogenic sprouting assays with DAPT treatment. Notch is a well characterized signalling pathway which plays a significant role in angiogenesis through the regulation of endothelial tip cell morphogenesis [41–45]. DAPT, (tert-butyl (2S)-2-[[(2S)-2-[[2-(3,5-difluorophenyl)acetyl]amino]propanoyl]amino]-2-phenylacetate), is an indirect Notch inhibitor through the direct inhibition of gamma-secretase. Notch inhibition is associated with the deregulation and increased formation of tip cells, resulting in larger and more numerous angiogenic sprouting in mouse retinal mount in vivo experiments.

In order to determine the morphological effects of DAPT induced Notch inhibition in angiogenic sprouting conditions, test groups were organized as follows: Untreated EGM-2 media, 0.25% DMSO vehicle control in EGM-2 media, and 25 µM DAPT with 0.25% DMSO vehicle in EGM-2 media. Imaged samples were processed via automated ImageJ macro script [38] to output mean vessel width, branch amounts, and mean vessel length. Untreated samples were designated as the reference by which the other two test group output values were normalized for comparison. In all morphological metrics observed, untreated reference vs vehicle control exhibited no significant differences (P > 0.05).

DAPT treatment vs control and reference exhibited thicker (1.214 vs 1 untreated, p = 1.7 × 10–10; vs 1.214 vs 0.981 DMSO, p = 2.7 × 10–10) vessels with more branches (1.177 vs 1 untreated, p = 1.466 × 10–5; 1.177 vs 1.049 DMSO, p = 0.049), but with similar vessel length (0.972 vs 1 untreated, p = 0.305; 0.972 vs 1.031 DMSO, p = 0.021).

DAPT induced increased vessel thickness and branching corroborates similar experiments utilizing murine in vivo retinal mount assays [46].

The demonstrated coupling of high throughput vascular tissue culture with automated high throughput vessel quantification shows promise as a powerful tool for the simultaneous study of multiple morphological metrics of vascular morphologies in a qualitative manner.

### Generation of perfusable vasculature for quantitative cancer intra/extravasation assays

**Fig. 5:**
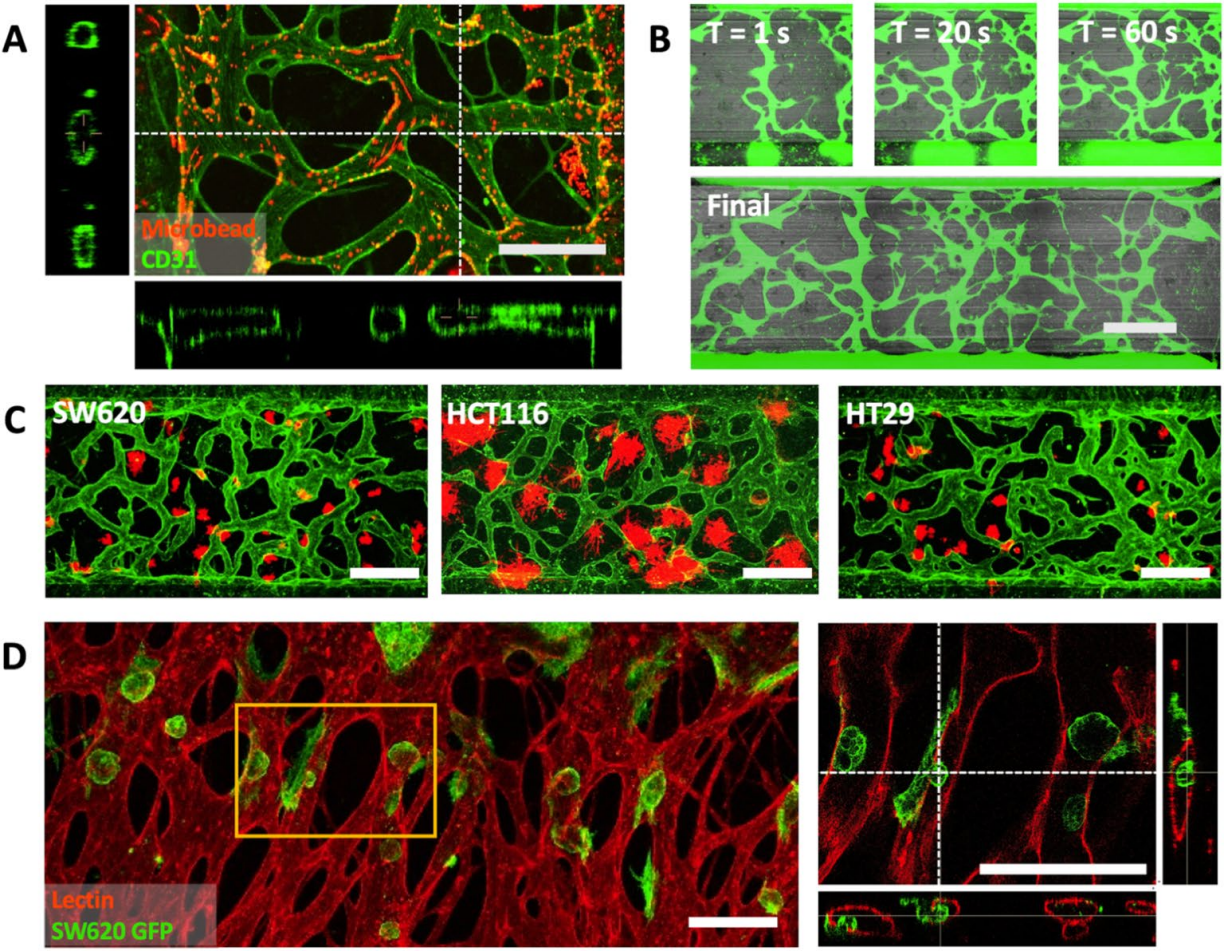
Overview of engineered end to end perfusable vasculature. **A** Confocal imaging of micro bead flow assay utilizing 2 µm beads through perfusable vasculature cultured within the chip. **B** Fluorescent dye assay showing FITC dye flow through the perfusable vasculature. **C** Confocal images of perfusable vasculature cultured with extravascular micro tumors. **D** Confocal images of intravascular micro tumors attached to and breaching the lumen. All scale bars are 500 µm

Engineered end-to-end perfusable vessel networks can serve as a potential basis for recapitulating sophisticated vascular networks to model more functional vascularized tissues [8, 47]. Perfusable vessel networks as encountered in vivo may yield a more thorough understanding of drug carrier penetration and transport through vasculature [48–50]. Perfusable vasculature (Fig. 5A) was generated by seeding a mixture of 6 × 10^6^ cells mL^−1^ HUVECs and 2 × 10^6^ cells mL^−1^ LFs in 2.5 mg mL^−1^ fibrinogen gel in the center channel, and seeding confluent monolayers of 6 × 10^6^ cells mL^−1^ HUVEC on both of the side channels. On day 0 (seeding), 100 µL of EGM-2 media was added to both reservoirs for a total of 200 µL and no flow. On day 1 and onwards, was completely aspirated and media flow was induced by adding 100 µL of fresh EGM-2 to one side and 50 µL to the other, alternating in direction daily until vessels are wide enough for the desired application. Vessels as shown in Fig. 5A were cultured with alternating flow into day 7 before fixation and bead assay. Perfusion was confirmed via confocal imaging of the vasculature using 488 conjugated CD31 to show that the endothelial cells formed tight junctions, as well as over-all lumen formation. 2 µm 594 conjugated micro-beads were then flowed through the vessel network by aspirating storage PBS from both reservoirs and adding 25 µL of diluted micro-bead suspension to one side.

With perfusable vasculature as a base platform, we generated two seeding configurations to model invasion of tumor cells from the extra luminal space to the lumen, and tumor cell extravasation from lumen to extra luminal space, both with a directional gradient of fresh and spent media through the vessel network. To model the growth of tumor cells within extravascular space and their interaction with and invasion into the vessel network (Fig. 5B), a tri-culture of HUVECs, LFs, and several colon cancer cell lines (SW620, HCT116, HT29) were seeded into the center channel between two confluent HUVEC monolayers and cultured using the perfusable vasculature protocol discussed previously. Each subtype showed qualitative differences in cluster morphology, with HTC116 showing wider and more loose dispersal of colonies, while SW620 and HT29 exhibited more compact micro tumors. To model the extravasation of circulating tumor cells (Fig. 5D), low concentration single-cell suspensions of SW620 were flowed through the vessel network by aspirating media from both reservoirs and adding a suspension of SW620 to one side, then cultured with flow. As shown, SW620 clusters formed from single circulating cells attached to the interior of the endothelial lumen, and breached the vascular lining into the extravascular space. Further work on quantitatively assaying cancer and vascular interactions with one another and to different treatment conditions utilizing this platform is currently underway.

## Conclusion

The MV-IMPACT platform is a powerful tool for robustly and repeatedly generating angio and vasculogenic assays, and for engineering perfusable vasculature. We applied the versatility and reliability of the platform to high throughput assays for stromal cell type and concentration dependent angiogenesis, for the screening of DAPT induced morphologies on angiogenic sprouts, and for the development of functional assays as a foundation for tumor invasion and circulating tumor extravasation.

Our design expands upon existing PDMS and injection molded microfluidic tissue culture platforms by utilizing novel patterning methods to reduce structural complexity and improve compactness, while maintaining robust patterning for consistent output. The 384 well plate duplex footprint of our platform occupies half of the space of a standard 96 well plate unit for double the throughput while preserving compatibility with multichannel pipettes and aspirators for hydrogel patterning and fluid handling, and decreasing hydrogel, cell, and media volumes needed per unit well (Additional file 1).

Utilizing high throughput droplet patterning, simultaneous multi-channel pipette mediated fluid handling, macro compatible image acquisition, and vessel imaging algorithms for high-throughput automated quantification of vessel morphologies, the MV-IMPACT combines the device accessibility of injection molded mass production with the experimental scalability, ease of use, and reliably consistent culture platform for a highly sensitive and robust basis for high content yet high throughput assays.

The MV-IMPACT improves upon the yield and scalability of sophisticated microfluidic tissue cultures, while simultaneously presenting an approachable system in terms of ease of use and device availability, as a much needed step towards wider scale implementation of organ-on-a-chip platforms.

## Supplementary Information

**Additional file 1.** Angiogenesis and vasculogenesis patterning configurations and sample output. **A**) Schematic diagram of an angiogenesis assay. 1μL of acellular fibrin gel is patterned in the central lane (red), followed by 2.5μL of lung fibroblasts in fibrin gel (green) in the right side lane, and 2.5μL of HUVEC suspension deposited as a confluent monolayer is seeded in the left (blue) side lane. Flow from the LF to EC compartments is induced by adding media to the LF side reservoir. Adjusted standard deviations for Area, Total Length, and Branches are: 0.1118, 0.0626, and 0.2047, respectively. **B**) Sample 28 well imaging output of angiogenesis within a single injection molded chip, center lane (red) was selected as ROI. **C**) Compares the sizes of a conventional PDMS based equivalent chip with the MV-IMPACT platform. An individual unit well of a PDMS chip is 22.5mm by 23.5mm, the MV-IMPACT platform is 4.5mm by 3.3mm – the equivalent of two 384 well microtiter plate wells, or half of a 96 well microtiter plate well. **D**) Shows a sample readout of an equivalent angiogenic assay on the 3 by 3 PDMS chip. Adjusted standard deviations for Area, Total Length, and Branches are: 0.3007, 0.2850, and 0.3090.

## Data Availability

The authors declare that the data supporting the findings of this study are available within the article and its supplementary information files.
