# Supplementary material for: Perfusable micro-vascularized 3D tissue array for high-throughput vascular phenotypic screening
Source: Nano Converg. 2022 Apr 8;9:16. doi: 10.1186/s40580-022-00306-w (PMC8994007; doi:10.1186/s40580-022-00306-w)
Supplement: Supplementary file 1 — Additional file 1. Angiogenesis and vasculogenesis patterning configurations and sample output. A) Schematic diagram of an angiogenesis assay. 1μL of acellular fibrin gel is patterned in the central lane (red), followed by 2.5μL of lung fibroblasts in fibrin gel (green) in the right side lane, and 2.5μL of HUVEC suspension deposited as a confluent monolayer is seeded in the left (blue) side lane. Flow from the LF to EC compartments is induced by adding media to the LF side reservoir. Adjusted standard deviations for Area, Total Length, and Branches are: 0.1118, 0.0626, and 0.2047, respectively. B) Sample 28 well imaging output of angiogenesis within a single injection molded chip, center lane (red) was selected as ROI. C) Compares the sizes of a conventional PDMS based equivalent chip with the MV-IMPACT platform. An individual unit well of a PDMS chip is 22.5mm by 23.5mm, the MV-IMPACT platform is 4.5mm by 3.3mm – the equivalent of two 384 well microtiter plate wells, or half of a 96 well microtiter plate well. D) Shows a sample readout of an equivalent angiogenic assay on the 3 by 3 PDMS chip. Adjusted standard deviations for Area, Total Length, and Branches are: 0.3007, 0.2850, and 0.3090. [file 40580_2022_306_MOESM1_ESM.docx]

Supplementary Materials


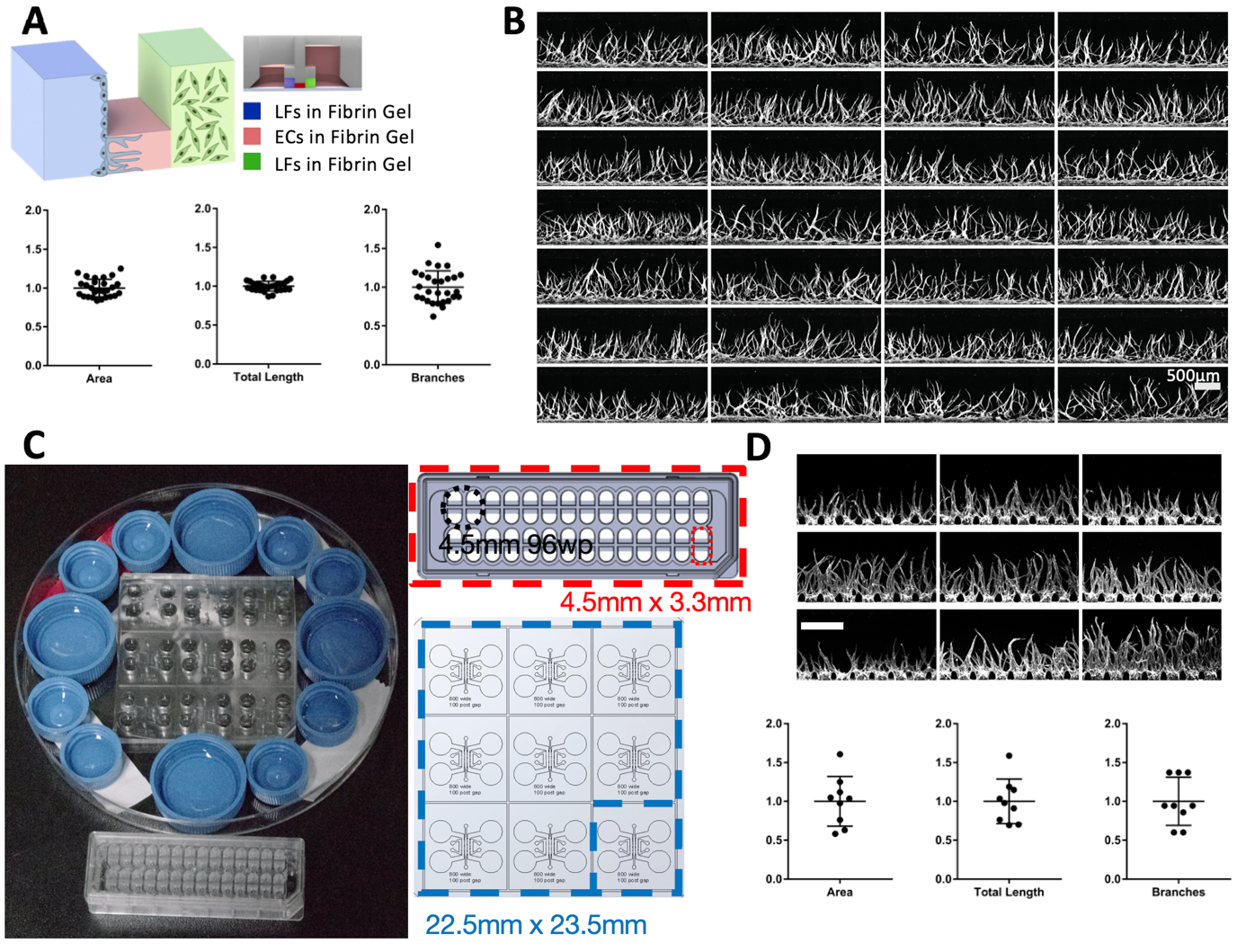


**Supplementary Fig. 1.** Overview angiogenesis and vasculogenesis patterning configurations and sample output. A) Schematic diagram of an angiogenesis assay. 1µL of acellular fibrin gel is patterned in the central lane (red), followed by 2.5µL of lung fibroblasts in fibrin gel (green) in the right side lane, and 2.5µL of HUVEC suspension deposited as a confluent monolayer is seeded in the left (blue) side lane. Flow from the LF to EC compartments is induced by adding media to the LF side reservoir. Adjusted standard deviations for Area, Total Length, and Branches are: 0.1118, 0.0626, and 0.2047, respectively. B) Sample 28 well imaging output of angiogenesis within a single injection molded chip, center lane (red) was selected as ROI. C) Compares the sizes of a conventional PDMS based equivalent chip with the MV-IMPACT platform. An individual unit well of a PDMS chip is 22.5mm by 23.5mm, the MV-IMPACT platform is 4.5mm by 3.3mm – the equivalent of two 384 well microtiter plate wells, or half of a 96 well microtiter plate well. D) Shows a sample readout of an equivalent angiogenic assay on the 3 by 3 PDMS chip. Adjusted standard deviations for Area, Total Length, and Branches are: 0.3007, 0.2850, and 0.3090.
